# Supplementary material for: Absorption/Attenuation Spectral Description of ESKAPEE Bacteria: Application to Seeder-Free Culture Monitoring, Mammalian T-Cell and Bacteria Mixture Analysis and Contamination Description
Source: Sensors (Basel). 2023 Apr 27;23(9):4325. doi: 10.3390/s23094325 (PMC10181643; doi:10.3390/s23094325)
Supplement: Supplementary file 1 [file sensors-23-04325-s001.zip › sensors-2263849-supplementary.pdf]

Supplementary Material of paper Sensors 2263849

Absorption spectra description of ESKAPEE bacteria: application to seeder-free culture monitoring, mammalian T-cell and bacteria mixture analysis and contamination description

Bruno Wacogne[1,2,*], Marine Belinger Podevin[1], Naïs Vaccari[1], Claudia Koubevi[1], Céline Codjiová[1], Emilie Gutierrez[1], Pauline Bourgeois[1], Lucie Davoine[1], Marjorie Robert-Nicoud[3], Alain Rouleau[1] and Annie Frelet-Barrand[1]

SD1 - Concerning spectroscopy cuvettes.

Basic plastic cuvettes were used and a large dispersion of their optical properties was observed principally due to a low fabrication reproducibility (figure S2). Absorption spectra were measured with 40 empty cuvettes using 1 extra cuvette for reference.

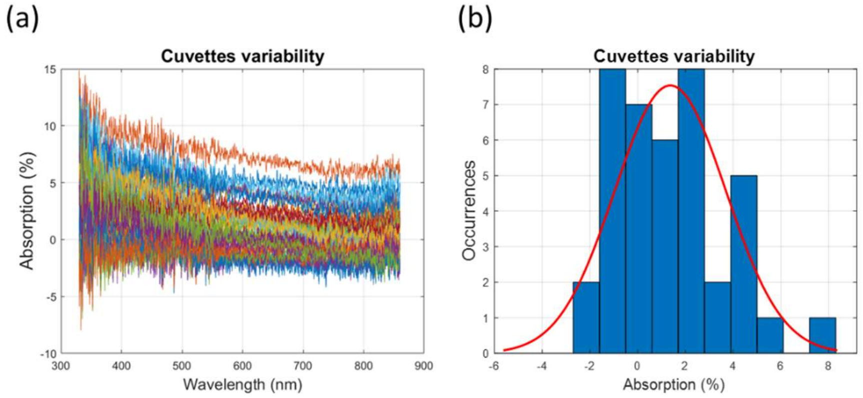

Figure S1: Variability of the spectroscopy cuvette optical properties. (a) Absorption spectra (n=40). (b) Histogram measured at 600 nm wavelength.

Statistically, the Full-Width-at-Half-Maximum of the histogram showed a variability of about ± 2.5%. In terms of *E. coli* concentration, this represented ± 2.5×10^{7} *E.coli*.mL^{-1} *i.e.* ± 25% at center concentration range or ±0.1 in log units. This approximately corresponds to dispersions mentioned in section 3.1.3. In figure S2, data were not exactly centered on 0 because the reference cuvette was absorbing slightly less than the average absorption of the 40 cuvettes.

SD2 - Extraction of the *E. coli* spectra.

The different steps of the extraction method are summarized in figure S1 for one spectrum. The raw spectra were truncated between 390 nm and 445 nm where the additional signal was observed (figure S1(a)). The truncated spectra were fitted with the *E. coli* function and subtracted from

the raw spectra to isolate the additional signals. The latter were smoothed using a Spline smoothing algorithm (figure S1(b)). The smoothed additional signals were then subtracted from the raw spectra to extract *E. coli* information (figure S1(c)).

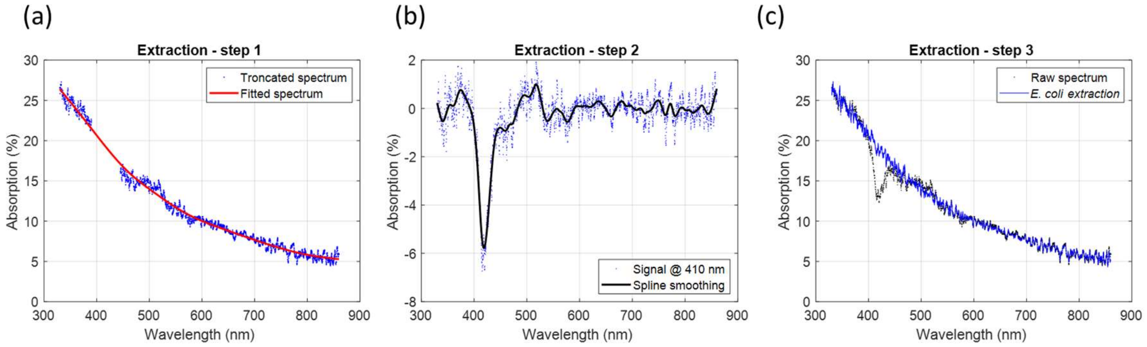

Figure S2: *E. coli* spectrum extraction. (a) Truncated spectrum fitted with the *E. coli* function. (b) Isolation and smoothing of the 410 nm signal. (c) Extracted *E. coli* spectrum.
